# Immune Markers and Tumor-Related Processes Predict Neoadjuvant Therapy Response in the WSG-ADAPT HER2-Positive/Hormone Receptor-Positive Trial in Early Breast Cancer

**DOI:** 10.3390/cancers13194884

**Published:** 2021-09-29

**Authors:** Nadia Harbeck, Raquel von Schumann, Ronald Ernest Kates, Michael Braun, Sherko Kuemmel, Claudia Schumacher, Jochem Potenberg, Wolfram Malter, Doris Augustin, Bahriye Aktas, Helmut Forstbauer, Joke Tio, Eva-Maria Grischke, Claudia Biehl, Cornelia Liedtke, Sanne Lysbet De Haas, Regula Deurloo, Rachel Wuerstlein, Hans Heinrich Kreipe, Oleg Gluz

**Affiliations:** 1 Breast Center, Department of Obstetrics and Gynecology and CCCLMU, University of Munich (LMU), Marchioninistrasse 15, 81377 Munich, Germany; Rachel.Wuerstlein@med.uni-muenchen.de; 2 The West German Study Group, 41061 Mönchengladbach, Germany; ronald.kates@t-online.de (R.E.K.); oleg.gluz@wsg-online.com (O.G.); 3 Evangelical Hospital Bethesda, 41061 Mönchengladbach, Germany; r.bailac@hotmail.com (R.v.S.); s.kuemmel@kem-med.com (S.K.); 4 Red Cross Hospital, 80634 Munich, Germany; michael.braun@swmbrk.de; 5 Breast Unit, Kliniken Essen-Mitte, 45136 Essen, Germany; 6 Klinik für Gynäkologie mit Brustzentrum Charité-Universitätsmedizin, 10117 Berlin, Germany; 7 St Elisabeth-Krankenhaus, 50935 Cologne, Germany; claudia.schumacher@hohenlind.de; 8 Evangelischen Waldkrankenhaus Spandau, 13589 Berlin, Germany; jochem.potenberg@jsd.de; 9 Breast Center, Department of Obstetrics and Gynecology, University Hospital Cologne, 50937 Cologne, Germany; brustzentrum-anmeldung@uk-koeln.de; 10 Breast Center, Clinic Deggendorf, 94469 Deggendorf, Germany; dorisaugustinprivat@yahoo.de; 11 University of Essen, 45147 Essen, Germany; Bahriye.Aktas@medizin.uni-leipzig.de; 12 Onkologie Rheinsieg, 53840 Troisdorf, Germany; forstbauer@onkologie-rheinsieg.de; 13 University Hospital Münster, 48149 Münster, Germany; tiojok@ukmuenster.de; 14 University Hospital of Tübingen, 72076 Tübingen, Germany; eva-maria.grischke@med.uni-tuebingen.de; 15 Westphalian Breast Center, City Hospital Dortmund, 44137 Dortmund, Germany; Claudia_biehl@yahoo.de; 16 University of Lübeck, 23562 Lübeck, Germany; cornelia1979@googlemail.com; 17 F. Hoffmann-La Roche Ltd., 4070 Basel, Switzerland; sanne_lysbet.de_haas@roche.com (S.L.D.H.); regula.deurloo@roche.com (R.D.); 18 Hannover Medical School, 30625 Hannover, Germany; Kreipe.Hans@mh-hannover.de

**Keywords:** biomarkers, breast cancer, HER2-positive, hormone receptor-positive, immune markers

## Abstract

**Simple Summary:**

Patients with “HER2-positive” early breast cancer are treated with antibodies to the HER2 protein along with chemotherapy, regardless of whether their cancer also has hormone receptors, or of its molecular features. Because patients with HER2-positive/hormone receptor-positive disease tend to live longer than those with HER2-positive/hormone receptor-negative disease, there may be some patients who are being overtreated under current guidelines. The aim of our exploratory translational analysis of the ADAPT HER2-positive/hormone receptor-positive trial was to investigate the potential of several prognostic (outcome regardless of therapy) and predictive (effect of therapy) biomarkers as early predictors of response to treatment before surgery. Comparison of these biomarkers before and after one treatment cycle, and their effects on whether patients’ cancers were completely removed at surgery, suggest that certain patients (those with treatment-induced CD8 protein-expressing cells infiltrating the cancer; without *PIK3CA* mutation; those with HER2-enriched tumors) may be candidates for less intensive treatment following pre-surgical therapy.

**Abstract:**

Prognostic or predictive biomarkers in HER2-positive early breast cancer (EBC) may inform treatment optimization. The ADAPT HER2-positive/hormone receptor-positive phase II trial (NCT01779206) demonstrated pathological complete response (pCR) rates of ~40% following de-escalated treatment with 12 weeks neoadjuvant ado-trastuzumab emtansine (T-DM1) ± endocrine therapy. In this exploratory analysis, we evaluated potential early predictors of response to neoadjuvant therapy. The effects of *PIK3CA* mutations and immune (CD8 and PD-L1) and apoptotic markers (BCL2 and MCL1) on pCR rates were assessed, along with intrinsic BC subtypes. Immune response and pCR were lower in *PIK3CA*-mutated tumors compared with wildtype. Increased BCL2 at baseline in all patients and at Cycle 2 in the T-DM1 arms was associated with lower pCR. In the T-DM1 arms only, the HER2-enriched subtype was associated with increased pCR rate (54% vs. 28%). These findings support further prospective pCR-driven de-escalation studies in patients with HER2-positive EBC.

## 1. Introduction

The current standard-of-care for HER2-amplified/-overexpressed (HER2-positive) early breast cancer (EBC) is anti-HER2 therapy plus chemotherapy, irrespective of hormone receptor (HR) status/molecular features. However, HER2-positive/HR-positive and HER2-positive/HR-negative EBC represent distinct entities. In HER2-positive EBC, evidence points to more favorable long-term survival in patients with HR-positive versus -negative disease [1], even among those receiving anti-HER2 therapy [2], with differences in key risk factors and distribution of first recurrence sites. Nevertheless, HR-positive disease is prognostically heterogeneous with respect to molecular subtypes [3,4,5], suggesting that it may be possible to identify subgroups that are candidates for de-escalated treatment. Specifically, in HER2-positive/HR-positive tumors, HER2 enrichment (by PAM50 signature) is far from universally prevalent, ranging from 17 to 55% [6,7,8,9]. The HER2-enriched PAM50 signature is associated with higher *HER2* expression and other 17q chromosome genes (e.g., *GRB7*), and lower *ESR1* expression [6,9]. Molecular heterogeneity within HER2-positive/HR-positive tumors is of particular interest in view of trials showing little (if any) benefit from adding trastuzumab (Herceptin,^®^ F. Hoffmann-La Roche Ltd., Basel, Switzerland) to chemotherapy among patients with low *HER2* copy numbers or intermediate *HER2* and high *ESR* expression [10,11].

The distinction between HER2-positive/HR-positive and HER2-positive/HR-negative EBC is reflected in differing pathological complete response (pCR) rates following neoadjuvant therapy and in the relative impacts of pCR on long-term survival [12,13].

De-escalation regimens are currently being investigated for both HER2-positive subtypes, aiming to decrease toxicity without compromising efficacy. In HER2-positive/HR-positive EBC, endocrine therapy (ET) plus anti-HER2 therapy (mostly dual anti-HER2 blockade) without systemic chemotherapy has been effective in the neoadjuvant setting [7,14]. Ado-trastuzumab emtansine (T-DM1; Kadcyla,^®^ F. Hoffmann-La Roche Ltd., Basel, Switzerland) is highly effective and well tolerated in the metastatic [15], neoadjuvant [16], and adjuvant settings (after standard therapy failure) [17]. Until recently, however, data regarding efficacy of single-agent T-DM1, or of T-DM1 plus ET, have been lacking, particularly in the neoadjuvant setting. The HER2-positive/HR-positive substudy of the three-arm, phase II–III Adjuvant Dynamic Marker-Adjusted Personalized Therapy Trial Optimizing Risk Assessment and Therapy Response Prediction in Early Breast Cancer (ADAPT) (NCT01779206) [18] has shown substantial pCR rates (no invasive tumor in the breast and lymph nodes) of ~40% after only 12 weeks in both T-DM1 study arms (with or without ET), compared to ~15% after trastuzumab plus ET.

In view of these encouraging findings and the known biologic heterogeneity of HER2-positive/HR-positive BC, patient selection for de-escalated (neoadjuvant) therapy is of key importance, motivating two central translational hypotheses. First, several lines of research suggest that biomarkers of immune response, apoptosis, and/or therapy resistance could be associated with pCR after neoadjuvant therapy—either prognostically, or predictively, regarding relative efficacy among potential regimens—and ultimately with long-term survival. Second, some biologic markers of response might emerge during the course of neoadjuvant therapy, pre-surgery. These and associated hypotheses are addressed below in the preplanned translational analysis of the neoadjuvant ADAPT HER2-positive/HR-positive trial. This manuscript focuses on pCR, which was the primary clinical endpoint of the trial and is also an important surrogate for survival in HER2-positive EBC [12].

## 2. Results

### 2.1. Patient and Sample Populations

**Figure 1:**
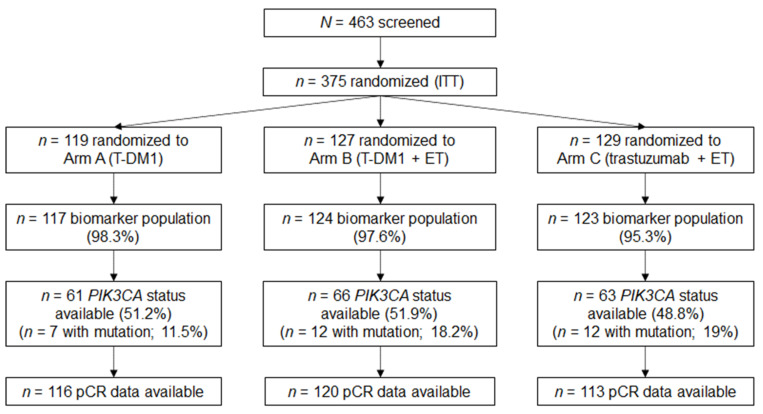
Patient disposition. ET, endocrine therapy; ITT, intent-to-treat; pCR, pathological complete response; T-DM1, ado-trastuzumab emtansine.

The primary pCR endpoint (yPT0 or ypT0is and ypN0) was assessable in 359/375 randomized patients (95.7%) [18]; pCR was observed in 48/117 patients (41.0%) treated by T-DM1, 51/123 (41.5%) of those treated by T-DM1 plus ET, and 18/119 (15.1%) of those treated by trastuzumab plus ET. Patient characteristics by arm were previously described [18]. The patient disposition for the current analysis is shown in Figure 1.

Evaluated biomarkers included *PIK3CA* mutation status, PAM50 gene expression levels and gene signature [19], and apoptosis (BCL2 and MCL1) and immune markers (programmed death-ligand 1 [PD-L1] and CD8) (Table 1). Characteristics of the baseline biomarker-evaluable populations were almost entirely representative of the intent-to-treat population [18]; tumor grade, progesterone receptor (PgR) status, estrogen receptor (ER) status, tumor dimension (pT), axillary lymph node status (pN), and menopausal status were similar between populations (aside from slightly lower prevalence of ER-negative receptor status in the *PIK3CA*-evaluable population). In contrast, as one might expect, the Cycle-2 biomarker-evaluable population comprised more patients with poorer response; in particular, a higher percentage from the trastuzumab arm. Negative PgR or ER receptor status was also less prevalent. The population with valid gene expression data comprised 253 patients, of whom 238 also had baseline biomarker data.

### 2.2. Biomarkers of Early Therapy Response

In immune cells (IC) and tumor cells (TC), PD-L1 scores were successfully assessed in 322/375 patients at baseline and 170/375 at Cycle 2 (Table 1). Subsequent analysis focused on IC, because at baseline, only 5/322 patients (<2%) had positive PD-L1 scores in TC and at Cycle 2 only 8/170 (<5%). Among patients with valid PD-L1–IC scores at baseline and Cycle 2, 23% had positive PD-L1–IC scores at baseline and 38% at Cycle 2, respectively (Figure S1). Paired Cycle 2 versus baseline PD-L1–IC scores were available in 151/375 cases (Table 1); under neoadjuvant therapy, PD-L1–IC scores increased in 28% and decreased in only 9% of paired cases (*p* < 0.001, McNemar test), with no significant differences by trial arm.

In addition to PD-L1, the T cell marker CD8 was assessed in baseline and Cycle 2 tumor samples. CD8/CNT-positivity was measured as percentage CD8+ cells in the tumor center, and CD8/INV-positivity was measured as percentage CD8+ cells in the invasive margin of the tumor. A total of 143 patients had paired CD8 evaluations in the tumor center (CD8/CNT); among these, CD8/CNT increased significantly among all patients and all three trial arms separately (all *p* < 0.001, Wilcoxon test). Although only 28 paired values were available for CD8 staining in the invasive margin of the tumor (CD8/INV), even this subset showed a significant (*p* = 0.003, Wilcoxon test) overall increase. Among patients with paired measurements, the mean increase of CD8 staining was ~100% in CD8/CNT (mean at baseline: 1.55; mean at cycle 2: 3.12) and ~85% for CD8/INV (mean at baseline: 1.28; mean at cycle 2: 2.37).

As for PD-L1–IC, the potential impact of CD8 as an early-response marker could be even higher than these results imply, due to missing data at Cycle 2 in samples with “low cellularity”.

A comparison of paired BCL2 and MCL1 H-Scores [20] showed that no significant changes in these antiapoptotic markers occurred in response to therapy.

### 2.3. Associations with Immune Biomarkers and Their Dynamic Changes with pCR

**Figure 2:**
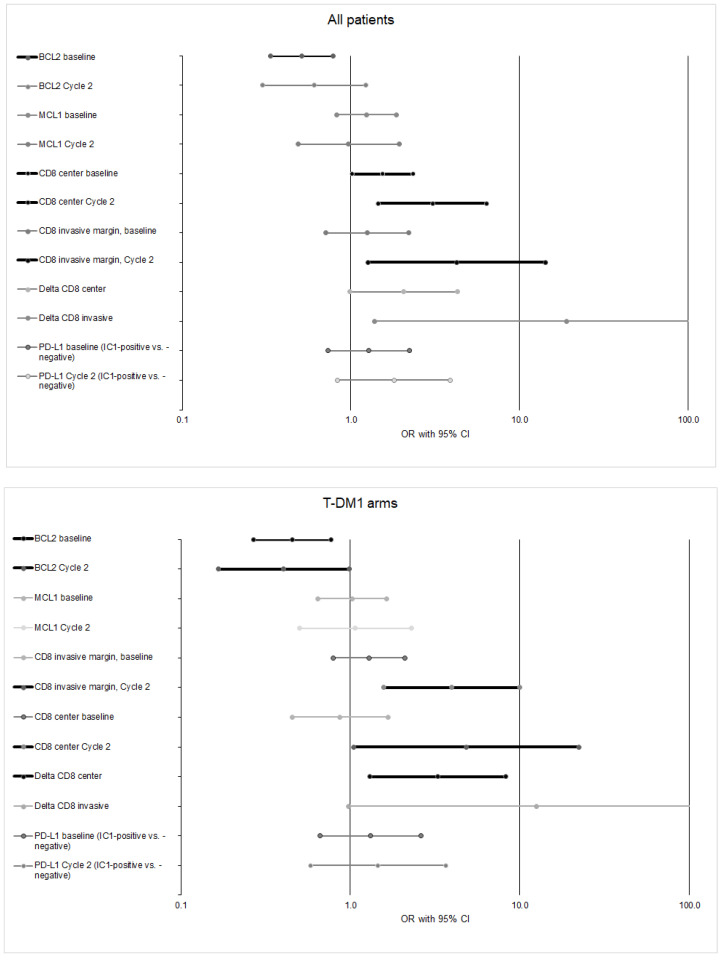
Unadjusted ORs of biomarkers at baseline and Cycle 2 for pCR in all patients (**top** panel) and in T-DM1 arms combined (**bottom** panel). All ORs are expressed as an interquartile ratio unless otherwise indicated (i.e., for PD-L1–IC). “Favorable” markers are those with OR >1. CNT, center; IC, immune cells; IC1, IHC staining in ≥1% and <5% of IC; INV, invasive margin; LCL, lower confidence limit; OR, odds ratio; PD-L1, programmed death-ligand 1; T-DM1, ado-trastuzumab emtansine; UCL, upper confidence limit.

Several tissue biomarkers had an impact on pCR (Figure 2). In all patients, baseline BCL2 (unfavorable), baseline and Cycle 2 CD8/CNT (favorable), Cycle 2 CD8/INV (favorable; limited sample size), and increases in either CD8/CNT or CD8/INV (favorable; limited sample size) all had significant or nearly significant impacts on pCR. The pattern was similar but not identical in the T-DM1 arms; besides baseline BCL2, Cycle 2 BCL2 was also negatively associated with pCR. Regarding PD-L1-IC (at baseline or Cycle 2), no association was found with pCR in all patients or the T-DM1 arms.

### 2.4. Impact of PIK3CA-Mutation Status on Early Therapy Response and pCR

*PIK3CA* mutation status was assessed in 190 patients: 177 at baseline, the rest at surgery (eight samples were available at both; all concordant). A total of 31/190 patients (16.3%) had mutations. There were no associations between mutation status and any baseline biomarker for PD-L1–IC or CD8/CNT (data not shown), nor with PAM50 classification (possibly due to low numbers with both available variables) (Figure S2).

**Figure 3:**
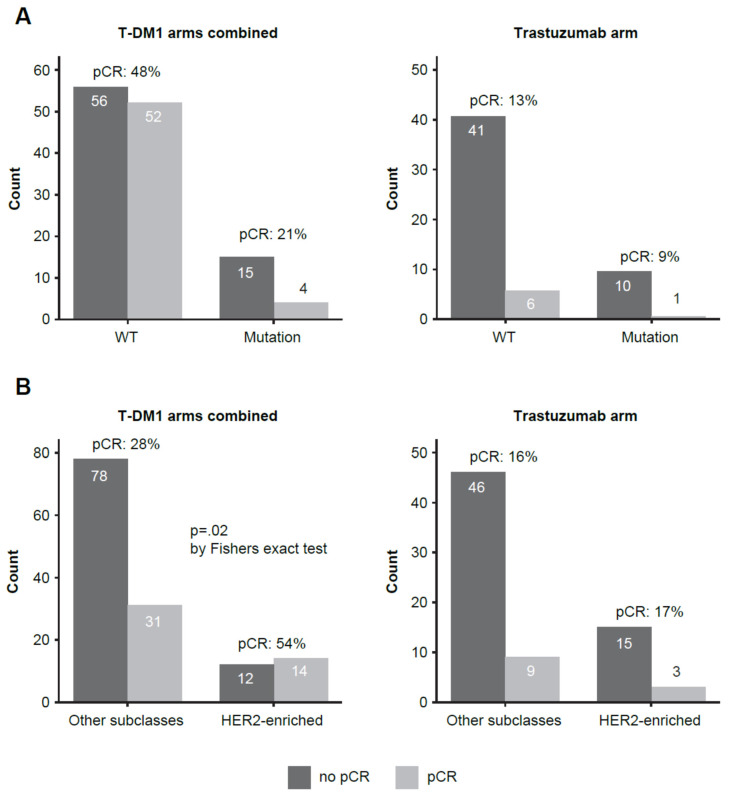
Overall pCR rates according to (**A**) *PIK3CA* mutation status and (**B**) in HER2-enriched tumors. *p* value calculated by Fisher’s exact test. pCR, pathological complete response; T-DM1, ado-trastuzumab emtansine; WT, wild-type.

Whereas CD8 protein expression generally increased following 3 weeks of therapy, and larger positive CD8/CNT responses (delta CD8/CNT, Figure 2) were themselves associated with pCR (particularly in the T-DM1-containing arms [*p* = 0.009]), CD8/CNT responses in *PIK3CA*-mutated tumors were small and lower than in wildtype (WT) tumors in all patients (*p* = 0.02) and separately in the T-DM1 arms (*p* = 0.01) (Figure S3). In line with observed poorer early response, overall pCR rates in *PIK3CA*-mutated tumors were only 16.7% versus 37.4% in WT samples (*p* = 0.04). A lower pCR rate among *PIK3CA*-mutated tumors (21.1% versus 48.1%) was separately observed in the combined T-DM1 arms (*p* = 0.04) (Figure 3A); the pCR rate for *PIK3CA*-mutated tumors was 9.1% versus 12.8% in the trastuzumab arm (*p* > 0.99).

### 2.5. Prevalence of PAM50 Intrinsic Subtypes and Their Association with pCR and Immune Markers

Valid PAM50 classification status was available in 350 samples: 187 at baseline; 136 at Cycle 2; 27 at surgery (Table 1). In 91 patients, valid PAM50 classification was available from samples taken at multiple timepoints; classifications were concordant in ~80% of these patients. Where samples were discordant, the earliest available sample yielding valid gene expression data and PAM50 classification was used. The resulting PAM50 intrinsic subtype classification was available in 215 patients: 118 (55%) luminal A; 49 (23%) luminal B; 46 (21%) HER2-enriched; and 2 (1%) basal-like.

In all patients, no significant association of individual PAM50 categories with pCR was seen (note that luminal A and luminal B are considered separate classes, and there were only two basal-like cases). Within the (pooled) T-DM1 arms, patients with HER2-enriched subtype had higher pCR than those with luminal or basal-like subtypes (54% to 28%, *p* = 0.02), but there was no advantage within the trastuzumab arm (17% vs. 16%) (Figure 3B), and in all patients there was only weak evidence of a difference (39–25%, *p* = 0.09).

HER2-enriched subtype showed a weak, but significant, positive association with higher baseline PD-L1 expression on IC, and higher CD8/INV expression at Cycle 2.

### 2.6. Association of Individual Gene Expression Levels with pCR

Underlying gene expression levels contributing to PAM50 classification were available for separate analysis. Unadjusted odds ratios of all 53 individual (standardized) gene expression levels for pCR were computed in each arm separately (Table 2).

In all patients, higher *ESR1*, *MAPT*, *CXXC5*, *SLC39A6*, and *PgR* levels were associated with lower pCR, while higher *HER2* (also known as *ERBB2*), *TMEM45B*, *GRB7*, and *RRM2* levels were associated with higher pCR. A similar tendency was seen for all these genes (except *PgR*) in the combined T-DM1 arms and the combined ET arms. Looking at individual arms, there were no direct examples of gene expression levels with an opposite (significant) odds ratio tendency (log odds >0 vs. <0) in different arms.

In order to gain insight into which genes might provide independent information, moderate-to-strong Spearman correlations (absolute values >0.4) among the genes appearing in Table 2 are listed in Table 3. In view of their strong correlation, one expects that *HER2* and *GRB7* are unlikely to be independent predictors of pCR, as investigated further by multivariable analysis. One observes that *CDC6* and *CENPF* do exhibit an opposing tendency in Arm A versus Arm B, despite their moderate positive correlation. Note that the genes associated with poorer pCR (*ESR1*, *MAPT*, *CXXC5*, *SLC39A6*, and *PgR*) in all patients are related to each other by moderate-to-strong correlations.

### 2.7. Association of HER2 Biomarkers with pCR

In view of the strong impact of *HER2* (*ERBB2*) expression by mRNA, the impact of immunohistochemical HER2 expression level (“IHC 3+” vs. lower) was also assessed: pCR was 45.7% for “IHC 3+” cases versus 12.9% for lower immunohistochemical HER2 expression (*p* < 0.001). The combination of HER2-enriched status with *HER2* mRNA expression did not improve prediction compared to HER2 alone.

### 2.8. Association of Individual Gene Expression Levels with Baseline Biomarkers and PIK3CA Mutations

None of the gene expression levels in Table 2 were associated with *PIK3CA* mutations, and there were no moderate (or strong) correlations of these genes with BCL2, MCL1, or PD-L1–IC baseline levels.

### 2.9. Association of Individual Gene Expression Levels with Immune Response

In view of the statistical associations of both immune response (marked by change in CD8/CNT) and of gene expression levels on pCR, a natural hypothesis is that the impact of gene expression levels could be at least partly mediated by the biologic process of immune response. In all patients with available data (*N* = 125), moderate (i.e., magnitude >0.25) Spearman correlations of CD8/CNT change existed for *CD8* and *PD-L1* gene expression (in the positive sense), and for *ACTR3B*, *ESR1*, *MLPH*, *BCL2*, *CXXC5*, *SLC39A6*, and *FOXA1* (in the negative sense). However, none of these correlations exceeded 0.35 in magnitude. Stepwise multiple regression analysis of CD8/CNT change on gene expression suggested that *CD8* (gene) and *KNTC2* were independent positive and that *CDH3* and *ESR1* were independent negative predictors of immune response.

### 2.10. Multivariable Models for Impact of Gene Expression Levels on pCR and Predictive Interactions

Multivariable logistic regression models to determine the impact of gene expression levels on pCR were computed as in the univariable analysis above in each arm separately, for all patients and in two subgroups: patients receiving T-DM1 (± ET), and patients receiving trastuzumab plus ET (Table 4). Remarkably, some gene expression levels that were not significant in univariable analyses emerged as significant in multivariable models.

The different genes entering the multivariable pCR models by therapy subgroup, particularly with respect to T-DM1 therapy, suggest the hypothesis of potentially predictive impacts, i.e., whether particular genes are associated with efficacy of T-DM1 compared with trastuzumab therapy. To test and quantify potential predictive impacts, an interaction analysis was performed (Table 4): all gene expression levels entering the multivariable models of Table 4 along with two therapy variables (T-DM1-containing vs. none and ET-containing vs. none) were tested as main effects (Table 5), as well as all therapy-gene expression interactions (Table 6).

The significant main effects were T-DM1 therapy in the neoadjuvant setting (favorable for pCR), and *GPR160* and *MIA* gene expression levels (both unfavorable at higher expression for any therapy arm). Higher *BAG1* under T-DM1 therapy had favorable impact on pCR, while higher *CXXC5* or *MAPT* essentially reduced the relative efficacy of the T-DM1 therapy arms (vs. the trastuzumab arm). *GRB7*, *KRT14*, and *RRM2* were independent favorable predictors of pCR under ET in the neoadjuvant setting (with trastuzumab/T-DM1).

## 3. Discussion

### 3.1. Trial De-Escalation

The WSG-ADAPT HER2-positive/HR-positive phase II neoadjuvant trial achieved pCR rates exceeding 40% after only 12 weeks of single-agent T-DM1 therapy ± ET versus ~15% with trastuzumab + ET [18]. By capturing biomarker data after three weeks of neoadjuvant therapy, the trial design has provided a unique opportunity to augment the information available at baseline with “early-response” biomarkers emerging during the course of neoadjuvant therapy. The preplanned translational analyses reported here, coupled with the substantial pCR rates in the T-DM1 arms, suggest that, by utilizing dynamic measurements of response to neoadjuvant therapy, it may soon be possible to distinguish patients with this disease entity who are candidates for de-escalation from those who require more aggressive therapy concepts.

### 3.2. Immune Response Mediates Efficacy of Anti-HER2 Therapy

The results taken together suggest a possible scenario for biologic response processes to neoadjuvant anti-HER2 therapy in a population with HER2-positive/HR-positive disease: one cycle of neoadjuvant anti-HER2-therapy (± ET) can induce an early immune response, marked here by tissue levels of CD8 and PD-L1, and potentially marked by tumor-infiltrating lymphocytes (TILs). Remarkably, immune response was induced even in the trastuzumab + ET arm. The immune response seems to mediate, though not entirely determine, the efficacy of anti-HER2 therapy, with much higher pCR under T-DM1 than trastuzumab. In line with this picture, baseline levels of the immune markers CD8 (a potential surrogate marker for cytotoxic tumor-infiltrating T-lymphocytes) and PD-L1 had a moderate predictive impact on pCR under anti-HER2 therapy, whether measured by IHC or by mRNA assessment. Notably, early immune response was evident in both CD8 protein (CNT or INV) and in PD-L1–IC. The key role of early immune response for pCR was evidenced most prominently by significantly higher pCR rates among patients with greater early (Cycle 2) CD8/CNT immune response, considered either as an independent marker or relative to the CD8/CNT baseline level, consistent with previous results implying an immune-modulating effect of T-DM1 [21].

Though early PD-L1 changes did not significantly predict higher pCR, a positive impact might have been masked by “missingness” of Cycle 2 PD-L1 scores due to “low cellularity”, which is itself a strong marker for early response and pCR [18]. Hence, the 28% rate of increased PD-L1–IC among paired cases, though substantial in itself, may even underestimate the potential impact of PD-L1–IC as an early therapy response marker.

It is noteworthy that these findings also strongly support a predictive combination model [22] utilizing “low cellularity” and TIL increases on treatment, rather than baseline assessment of immune infiltrate, as markers for early efficacy estimation of de-escalated anti-HER2 treatment.

Some challenges remain to be addressed in utilizing immune response as a predictor in HER2-positive/HR-positive breast cancer: several (but not all [23]) neoadjuvant trials in HER2-positive EBC have shown a strong predictive impact of immune markers (CD8 expression and/or TILs) on pCR [24,25,26]. Immune infiltrate measured by IHC (e.g., CD8, programmed death-1 expression) or by TILs are considered as predictors for higher pCR, higher efficacy for adjuvant pertuzumab (PERJETA^®^, F. Hoffmann-La Roche Ltd.) addition to trastuzumab [4] and better prognosis in HER2-positive EBC [24,27]; however, these results are somewhat controversial regarding pCR prediction after docetaxel plus pertuzumab plus trastuzumab within the NeoSphere trial in HER2-positive/HR-negative disease [23]. Moreover, the impact of immune response appears less well-established with the HR-positive subgroup of HER2-positive disease [25]. Although a strong correlation has been observed between higher TIL levels and the eight-gene trastuzumab response signature, no predictive effect of TILs on trastuzumab survival benefit in the adjuvant setting has been reported so far [27]. Remarkably, dynamic change in immune response (e.g., change of TILs during therapy vs. baseline TIL levels) seems to be more predictive for efficacy of de-escalated treatment, in particular [22]. Finally, the optimal measurement method for functional immune infiltrates (TILs vs. IHC vs. immune signature) in breast cancer remains unclear [25,28].

### 3.3. PIK3CA

The present translational analysis revealed that immune response and pCR were lower in *PIK3CA*-mutated tumors than in WT, independent of all other factors. Mutation status has been previously associated with poorer prognosis, particularly in HER2-positive/HR-positive disease [29], irrespective of molecular subtype, and mutation is considered as a candidate marker for resistance to anti-HER2 treatment in the neoadjuvant setting [29,30], with or without chemotherapy [31].

Response to therapy containing anti-HER2 and/or antihormonal agents in the neoadjuvant setting appears to be a highly multifactorial process; mutation, present in approximately 17% of patients in this population, seems to constitute a resistance marker to all therapies in this trial. These findings are consistent with lower pCR in tumors with mutation treated by six cycles of T-DM1+ pertuzumab (31% vs. 51%) in an unselected HER2-positive cohort from the KRISTINE trial [32]. Remarkably, docetaxel, carboplatin, trastuzumab, and pertuzumab were superior to T-DM1 (plus pertuzumab) only in patients with mutation. Similar to the KRISTINE trial, we see lower HER2 expression levels by mRNA and/or IHC in T-DM1-treated patients with mutation [32], which could be an explanation. In contrast to other studies [5], no significant association of mutation status with luminal subtype was seen here. Our results in EBC are in contrast with data from the EMILIA trial, in which mutations had no impact on T-DM1 efficacy (in unselected HER2-positive MBC) [33]. More recently, mutation was found to be associated with worse clinical outcome in an exploratory analysis of the MARIANNE study [34]; hence, the impact of mutation status on T-DM1 efficacy in the MBC setting remains unclear.

Most neoadjuvant studies have reported lower pCR in *PIK3CA*-mutated cases treated by single or double anti-HER2 blockade with or without chemotherapy [29,30,31]. For the first time, we have observed reduced immune response as characterized by change in CD8 protein in the tumor center, even under T-DM1 therapy, although T-DM1 may still be more effective than trastuzumab in mutated tumors. Although recent prognostic as well as predictive data regarding efficacy of adjuvant anti-HER2 therapy according to mutation status are controversial [4,5,35], the evidence would favor focusing current concepts of de-escalation on patients with WT status [31], while evaluating novel approaches in patients with mutated tumors.

### 3.4. Results on PAM50 and Genes

The current translational analysis included determination of PAM50 subtypes and evaluation of their impacts on response. Our luminal-subtype incidence of ~78% is somewhat higher than reported in HER2-positive/HR-positive disease (~50%) [9,36], but in line with other studies [6,25,37]. Compared to other subtypes (luminal and basal), HER2-enriched subtype (found in ~20% of patients) was associated with approximately double the pCR rate in the T-DM1 arms (54% vs. 28%) but afforded no advantage in the trastuzumab arm. These results are in line with KRISTINE [37].

Although no clear prognostic impact of PAM50 subtypes and/or benefit from anti-HER2 treatments have been reported in HER2-positive/HR-positive EBC [3,4,5,36,38,39], all neoadjuvant trials involving chemotherapy with single or dual anti-HER2 blockade show significantly higher pCR rates in patients with HER2-enriched HER2-positive/HR-positive tumors (~40% and 54–63% by mono- and polychemotherapy, and 32–45% after chemotherapy-free regimens, respectively). Lower pCR rates of ~30% after chemotherapy and ~10% after chemotherapy-free anti-HER2-based regimens were observed in luminal subtypes in most trials [6,7,9,36].

These considerations suggest that determination of HER2-enriched subtype may be useful for selecting patients for anti-HER2-based pCR-directed de-escalation. However, although HER2-enriched subtype was associated with higher pCR and with other factors such as HER2 (higher expression), ESR1, PGR, and BCL2 (by IHC), PAM50 subtype does not seem to capture all of the prognostic information encoded in individual gene expression levels. In our trial, individual gene expression levels showed prognostic impact on pCR, part of which may be mediated by immune response, as well as hints of predictive impact regarding neoadjuvant therapy. Interaction analysis revealed that certain genes may be associated with relative efficacy of T-DM1 versus trastuzumab or of addition of ET to anti-HER2 therapy versus no ET. Furthermore, the results of interaction analysis for pCR appear to be broadly consistent with multivariable subgroup analyses in the current translational analysis.

In the whole cohort, higher expression of single genes such as *HER2* and *GRB7* (which is strongly associated with *HER2*) and/or lower expression of *ESR1*, *PgR*, and others impacted pCR independently of molecular subtyping. The present results are consistent with previous findings, such as identification of the combination (HER2-enriched subtype and *HER2*-high status) as a marker for enhanced benefit from chemotherapy-free, anti-HER2 regimens [9]. Studies have revealed HER2-enriched subtype and high *HER2* expression as predictors for higher pCR after standard or de-escalated regimens [9]. The predictive impact of PAM50 subtypes on trastuzumab benefit in the adjuvant setting was less pronounced [38,39].

In the current translational analysis, *CD8* (by gene expression) and *KNTC2* were independent positive predictors, and *CDH3* and *ESR1* were independent negative predictors of immune response, characterized here by change in CD8/CNT (in patients with paired CD8 measurements).

Regarding the impact of HER2-enriched subtype, further research is strongly needed in light of our results as well as limited, if any, benefit from anti-HER2 treatment in the neoadjuvant–adjuvant settings in patients with high ESR and low HER2 expression [4,6,10,11], particularly in molecularly heterogeneous HER2-positive/HR-positive breast cancer. An optimal method for identification of “HER2-sensitive” disease should be addressed by prospective trials prior to recommendations of routine use of gene sequencing tools in HER2-positive EBC. Future trials should address the relative merits of multigene-based profiles versus immunohistochemical HER2 expression levels (“IHC 3+” vs. “IHC 2+”) or high *HER2* copy numbers, which are predictive markers for pCR of ≥50% following T-DM1 therapy in the current trial and/or in other trials [32,40].

Expression levels of BCL2 have been reported to vary across molecular subtypes in BC, with expression significantly associated with low proliferative factors and HR positivity [41]. In this analysis, higher levels of BCL2 at baseline were associated with lower pCR in all patients; in the T-DM1 arms (combined), the impact of higher BCL2 at either baseline or Cycle 2 on pCR was also unfavorable, and if anything, even more pronounced, consistent with the interpretation of BCL2 as a potential marker for resistance to T-DM1 therapy.

### 3.5. Limitations

While translational research in the prospective neoadjuvant WSG-ADAPT HER2-positive/HR-positive neoadjuvant trial was pre-planned, the specific analyses performed were planned after the protocol was approved. As reported above, the Cycle 2 biomarker-evaluable population was not representative of the baseline population (more patients had poorer response; there was a higher percentage from the trastuzumab arm; fewer patients had PgR- or ER-negative receptor status). Statistical *p*-values were not corrected for multiple testing, and results, particularly predictive impacts, are considered exploratory and hypothesis-generating only.

## 4. Conclusions

In conclusion, the neoadjuvant WSG-ADAPT HER2-positive/HR-positive trial has demonstrated that de-escalation is possible in HER2-positive/HR-positive EBC, with a very promising pCR after only four cycles of T-DM1, particularly in selected cohorts, e.g., with treatment-induced CD8 immune infiltrate and/or *PIK3CA* wildtype and/or HER2-enriched and/or high *HER2* and lower *ESR1*/*MAPT* expression. Induction of immune response by T-DM1, as shown previously [21], suggests a combination of immunotherapy and T-DM1, at least in patients without *PIK3CA* mutation, to enhance efficacy. These findings are of particular interest together with similar pCR [42] and invasive disease-free survival [43] data in the PREDIX and KRISTINE trials, but substantially better safety favors the T-DM1 arm. Furthermore, a numerical benefit in terms of OS was observed for T-DM1 in combination with atezolizumab versus T-DM1 plus placebo in the PD-L1–IC+ subgroup of the KATE2 study in advanced BC [44]. As previously reported [45], CD8 expression and CD8 dynamics were more strongly predictive for efficacy than TILs, but very high baseline TIL levels (>40%) were associated with an excellent pCR rate of 70% in patients treated with T-DM1.

At present, we would consider patients with HER2-positive EBC and CD8+ infiltrate and/or TILs at baseline and/or after one cycle of anti-HER2 treatment as possible candidates for a de-escalated (chemotherapy) approach, based on their favorable prognosis [4,24,27] (particularly in the HER2-positive/HR-positive cohort [1,46]). Beyond patient selection, de-escalation therapy needs to be further optimized in prospective trials in view of the conflicting data regarding optimal duration and combination of anti-HER2 treatment presented by ShortHER [47] and APHINITY [4]. Nevertheless, our findings strongly support further prospective, pCR-driven, antibody–drug conjugate-based de-escalation concepts in carefully selected patients with HER2-positive EBC, particularly in HER2-positive/HR-positive disease.

## 5. Materials and Methods

### 5.1. Patients and Trial Design

The trial design (Figure S4) has been described previously [18].

Briefly, patients had tumors that were ER-positive and/or PgR-positive and HER2-positive by central pathology confirmation, and they had cT1c to cT4a–c and any cN disease, no clinical evidence of distant metastases (M0), adequate organ function, and left ventricular ejection fraction ≥50% within normal institutional limits by echocardiography with a normal electrocardiogram. A total of 375 patients were randomized: 119 to T-DM1; 127 to T-DM1 plus ET; 129 to trastuzumab plus ET. Recommended ET consisted of tamoxifen for premenopausal women and aromatase inhibitors for postmenopausal women. Post-surgery, patients received standard therapy: four epirubicin and cyclophosphamide cycles (all patients) followed by 12 weekly paclitaxel doses (patients treated with trastuzumab and ET), 40 weeks of trastuzumab, radiotherapy (if indicated), and ET. Postoperative (adjuvant) chemotherapy was mandatory for patients with non-pCR, but was optional for patients with pCR.

The trial is conducted in accordance with the Declaration of Helsinki, ICH-GCP, and all applicable laws and requirements. The trial received approvals from the institutional ethics committee (University of Cologne; protocol code WSG-AM06; date of approval 2 July 2015) and informed consent, including for blood and tissue sample donation, was obtained from all patients of the ADAPT trial and its substudies [48].

### 5.2. pCR Assessment

Tumor samples were assessed for the primary endpoint, pCR, by local pathology review of samples taken at surgery following the completion of neoadjuvant therapy.

### 5.3. Biomarker Assessment

Immune markers were assessed by IHC of formalin-fixed, paraffin-embedded tumor samples from three timepoints: in core biopsies at baseline and Cycle 2, and in a smaller number of samples at surgery. Most of the analyses reported here pertain to the first two timepoints.

CD8 staining was performed using clone C8/144B on the Ventana Benchmark XT platform (Ventana Medical Systems, Inc., Tucson, AZ); CD8/CNT-positivity (measured as percentage in tumor center) and CD8/INV-positivity (measured as percentage in the invasive margin) were coded as percentage of positively stained cells. CD8 change was defined as Cycle 2 minus baseline value.

Staining for antiapoptotic markers BCL2 (invasive tumor cells) and MCL1 was performed using clones 124 and SP143 (Ventana Medical Systems, Inc., Tucson, AZ), respectively, on the Ventana Benchmark XT platform; for BCL2 expression, positivity on lymphocytes served as an internal control. H-Scores for antiapoptotic markers BCL2 and MCL1 were assessed at baseline and Cycle 2 [20].

Staining for PD-L1 by IHC utilized the VENTANA SP142 antibody (research use only) on the Ventana Benchmark XT platform. PD-L1-positivity on IC was determined by the proportion of positively stained tumor area, while PD-L1-positivity on TC was determined by the percentage of positively stained TC. PD-L1-positivity (IC and TC) was defined as PD-L1-positive staining in ≥1% of the tumor area/TC.

Hematoxylin and eosin evaluation was performed by a certified pathologist to assist in the interpretation of the CD8 and PD-L1 IHC analyses.

High-throughput microfluidic quantitative polymerase chain reaction (MUT-MAP 13-gene panel) was used to assess *PIK3CA* mutations [49].

Core-cut biopsies were obtained at baseline as part of routine diagnostic work-up, as well as after 3 weeks of chemotherapy (as part of a translational protocol). For stromal TIL (sTIL)-analysis, formalin-fixed and paraffin-embedded tissue was cut at 4–5 μm thickness and transferred to slides. Staining was performed using hematoxylin-eosin. Slides were digitalized using the Aperio ImageScope 12.0 software (Leica, Germany, Version 12.3.0.5056) and analyzed both qualitatively and quantitatively at 200–400× magnification. In accordance with international guidelines, we confined our analysis to quantification of sTILs. sTIL counts were quantified in relation to surrounding tumor tissue as previously recommended [50]. sTIL infiltrates in tumor-surrounding normal breast tissue as well as in ductal carcinoma in situ tissue were excluded, as were necrotic or fibrotic areas, cell-free sclerosis, areas of florid granulocytic inflammation, and extensive regressive hyalinization.

Gene expression (RNA) was assessed by a custom 800-gene codeset on the nCounter platform (Nanostring Technologies, Inc., Seattle, WA, USA) on all baseline and Cycle 2 biopsy samples. The panel of genes included those required to assess intrinsic breast cancer subtypes according to PAM50 [19]. PAM50 subtypes (HER2-enriched, luminal A, luminal B, and basal-like) were assigned by the random-forest-based classifier [51].

### 5.4. Statistical Analysis

The biomarker analyses reported here were preplanned but exploratory in nature; *p*-values were not corrected for multiple testing.

Associations among nominal variables were assessed by Fisher’s exact test. The McNemar test was used to compare paired ordinal scores. The Wilcoxon test was used to compare paired scaled variables. Associations of continuous variables (including individual genes) with pCR were analyzed by univariable and multivariable (stepwise) logistic regression to compute unadjusted and adjusted odds ratios, respectively; these analyses were carried out in all patients and in neoadjuvant therapy subsets. To facilitate quantitative evaluation of effect sizes, expression levels of individual genes were standardized (transformed to zero mean and unit standard deviation) for inclusion in logistic regression; thus, “standardized odds ratios” refers to a one-standard-deviation increment. Other continuous variables, including PD-L1–IC, PD-L1–TC, CD8/CNT, CD8/INV, and their changes between baseline and Cycle 2, were coded by fractional ranks in the population; odds ratios associated with fractionally ranked variables correspond to the interquartile range (75th vs. 25th percentile). Upper and lower 95% (uncorrected) confidence limits (UCL and LCL, respectively) are reported. Gene expression variables significant in multivariable prognostic models for pCR were entered into an interaction analysis, including therapy variables as main effects, as well as all therapy–gene interactions. Spearman correlations were computed to quantify the joint distribution of key gene expression variables and to characterize potential associations of immune response indicators with gene expression and other baseline measurement; linear regression was also carried out to investigate the impact of gene expression on continuous variables emerging as markers of response.

## Figures and Tables

**Table 1:** Availability of biomarkers.

Biomarker	*N* Composite, *n* (%)	Baseline, *n* (%)	Cycle 2, *n* (%)	Paired Cycle 2 vs. Baseline, *n* (%)	At Surgery, *n* (%)
PD-L1–IC	–	322 (86)	170 (45)	151 (40)	21 (6)
PD-L1–TC	–	322 (86)	170 (45)	151 (40)	21 (6)
CD8/CNT	–	313 (83)	166 (44)	143 (38)	20 (5)
CD8/INV	–	162 (43)	60 (16)	28 (7)	10 (3)
BCL2	–	321 (86)	168 (45)	151 (40)	23 (6)
MCL1	–	325 (87)	169 (45)	150 (40)	25 (7)
PAM50	215 (57)	187 (50)	136 (36)	–	27 (7)
*PIK3CA* mutation status	190 (51)	177 (47)	–	–	21 (6)

Abbreviations: CNT, center; IC, immune cells; INV, invasive margin; PAM50, Prediction Analysis of Microarray 50.

**Table 2:** Unadjusted ORs of (standardized) gene expression measurements for pCR (with 95% confidence intervals) by univariable logistic regression (genes with significant impact). ORs ≤ 1 are shown in red; ORs > 1 are shown in black.

**Gene**	**Arm A: T-DM1** **(*n* = 76)**				**Arm B: T-DM1 + ET** **(*n* = 82)**				**Arm C: Trastuzumab + ET** **(*n* = 82)**			
	**OR**	**LCL**	**UCL**	***p***	**OR**	**LCL**	**UCL**	***p***	**OR**	**LCL**	**UCL**	***p***
*ACTR3B*	0.48	0.24	0.96	0.04								
*CDC6*	0.55	0.32	0.93	0.03								
*CENPF*					2.02	1.10	3.74	0.02				
*CXXC5*	0.47	0.26	0.84	0.01								
*HER2*					2.30	1.31	4.03	0.004	2.10	0.99	4.44	0.05
*ESR1*	0.48	0.27	0.84	0.01								
*GRB7*					2.27	1.35	3.83	0.002	2.26	1.08	4.77	0.03
*MAPT*	0.54	0.31	0.94	0.03	0.57	0.35	0.94	0.03				
*MIA*	0.50	0.28	0.88	0.02								
*PgR*	0.56	0.33	0.95	0.03								
*RRM2*					1.78	1.05	3.01	0.03				
*TMEM45B*					1.78	1.06	2.99	0.03				
**Gene**	**Entire Gene Expression Population with pCR Endpoint (*n* = 240)**				**T-DM1 Arms (A and B) Only (*n* = 158)**				**ET arms (B and C) Only** **(*n* = 164)**			
	**OR**	**LCL**	**UCL**	***p***	**OR**	**LCL**	**UCL**	***p***	**OR**	**LCL**	**UCL**	***p***
*CENPF*					1.52	1.03	2.24	0.03				
*CXXC5*	0.70	0.52	0.95	0.02	0.59	0.40	0.87	0.01				
*HER2*	1.76	1.27	2.44	0.001	1.78	1.22	2.60	0.003	2.08	1.35	3.21	0.001
*ESR1*	0.64	0.48	0.85	0.002	0.58	0.40	0.84	0.004	0.70	0.49	1.00	0.05
*FGFR4*					1.40	1.00	1.96	0.05				
*GRB7*	1.84	1.34	2.53	<0.001	1.79	1.25	2.56	0.002	2.20	1.45	3.35	<0.001
*MAPT*	0.63	0.47	0.84	0.002	0.56	0.39	0.80	0.002	0.66	0.47	0.94	0.02
*PgR*	0.74	0.56	0.99	0.04								
*RRM2*	1.52	1.11	2.08	0.01	1.63	1.11	2.40	0.01	1.50	1.02	2.19	0.04
*SLC39A6*	0.68	0.50	0.93	0.02	0.70	0.48	1.00	0.05	0.63	0.43	0.94	0.02
*TMEM45B*	1.38	1.01	1.89	0.05	1.47	1.03	2.10	0.03	1.59	1.04	2.43	0.03

The relative impacts on pCR in the table are directly comparable because the ORs are expressed with respect to a one-standard-deviation change in the corresponding gene expression value. The ORs reported are those retained in the univariable logistic regression models. Abbreviations: ET, endocrine therapy; LCL, lower confidence limit; OR, odds ratio; T-DM1, ado-trastuzumab emtansine; UCL, upper confidence limit.

**Table 3:** Spearman correlations exceeding 0.4 among gene expression measurements of Table 2.

Spearman Correlations		
*CDC6*	*CENPF*	0.45
*CENPF*	*RRM2*	0.53
*CXXC5*	*ESR1*	0.48
*HER2*	*GRB7*	0.85
*ESR1*	*MAPT*	0.46
*MAPT*	*PgR*	0.69
*ESR1*	*SLC39A6*	0.57

**Table 4:** Adjusted standardized ORs of genes with significant impact on pCR in multivariable logistic regression models. All 53 gene expression levels and PAM50 subtypes were entered. ORs ≤ 1 are shown in red; ORs > 1 are shown in black.

**Multivariable pCR Models**	**Arm A: T-DM1** **(*n* = 76)**				**Arm B: T-DM1 + ET** **(*n* = 82)**				**Arm C: Trastuzumab + ET (*n* = 82)**			
**Gene**	**OR**	**LCL**	**UCL**	***p***	**OR**	**LCL**	**UCL**	***p***	**OR**	**LCL**	**UCL**	***p***
*BAG1*	5.11	1.81	14.38	0.002								
*BLVRA*					0.38	0.16	0.95	0.04				
*CDC6*	0.39	0.18	0.84	0.02								
*CXXC5*	0.42	0.18	0.94	0.03								
*FOXC1*					2.26	1.19	4.29	0.01				
*GPR160*	0.34	0.15	0.78	0.01								
*GRB7*					3.49	1.62	7.52	0.001	2.26	1.08	4.77	0.03
*MIA*	0.42	0.19	0.90	0.03								
*MMP11*					0.40	0.20	0.83	0.01				
*PgR*	0.31	0.15	0.66	0.002								
*RRM2*					1.94	1.01	3.73	0.05				
**Multivariable pCR Models**	**Entire Gene Expression Population with pCR Endpoint (*N* = 240)**				**T-DM1 Arms (A and B) Only (*n* = 158)**				**ET Arms (B and C) Only** **(*n* = 164)**			
**Gene**	**OR**	**LCL**	**UCL**	***p***	**OR**	**LCL**	**UCL**	***p***	**OR**	**LCL**	**UCL**	***p***
*BAG1*	1.55	1.08	2.23	0.02	1.82	1.15	2.88	0.01				
*CDC6*	0.72	0.51	1.00	0.05								
*CDH3*									0.54	0.32	0.91	0.02
*CXXC5*					0.54	0.36	0.82	0.004				
*HER2*					1.66	1.10	2.51	0.02				
*FOXA1*	0.60	0.42	0.84	0.003								
*GRB7*	2.08	1.45	2.98	<0.001					2.54	1.60	4.03	<0.001
*KRT14*									2.77	1.45	5.27	0.002
*MAPT*					0.51	0.33	0.78	0.002				
*RRM2*	1.59	1.10	2.31	0.01								
*SLC39A6*									0.49	0.29	0.84	0.01

Abbreviations: ET, endocrine therapy; LCL, lower confidence limit; OR, odds ratio; pCR, pathological complete response; T-DM1, ado-trastuzumab emtansine; UCL, upper confidence limit.

**Table 5:** Adjusted standardized ORs of genes, including therapy variable, with significant impact on pCR in multivariable logistic regression models. All 53 gene expression levels and PAM50 subtypes were entered. Significant ORs ≤ 1 are shown in red; ORs > 1 are shown in black. Abbreviations as above.

Multivariable pCR Including Therapy	Entire Gene Expression Population with pCR Endpoint (*N* = 240)			
Factor	OR	LCL	UCL	*p*
T-DM1 therapy	3.06	1.51	6.21	0.002
*FOXA1*	0.61	0.43	0.85	0.004
*GRB7*	2.07	1.44	2.99	<0.001
*RRM2*	1.46	1.02	2.10	0.041

**Table 6:** Multivariable logistic regression models to predict pCR including therapy, all genes (RNA-expression) with significant impact analyzed, and all gene-therapy interactions.

Multivariable Interaction pCR Models	Entire Gene Expression Population with pCR Endpoint (*N* = 240)			
Factor	OR	LCL	UCL	*p*
T-DM1 therapy (either arm)	3.60	1.63	7.98	0.002
*GPR160*	0.63	0.41	0.95	0.03
*MIA*	0.65	0.43	1.00	0.05
*BAG1* by T-DM1 therapy	2.42	1.43	4.09	0.001
*CXXC5* by T-DM1 therapy	0.49	0.30	0.78	0.003
*MAPT* by T-DM1 therapy	0.49	0.31	0.78	0.002
*GRB7* by ET	2.39	1.48	3.87	<0.001
*KRT14* by ET	2.78	1.46	5.31	0.002
*RRM2* by ET	1.97	1.13	3.43	0.02

(ET = endocrine therapy, i.e., either T-DM1 + ET or trastuzumab + ET arm). All ORs involving genes, including the interaction terms, refer to a one-standard-deviation increase in the gene expression variables (see Section 5.4). ORs ≤ 1 are shown in red; ORs > 1 are shown in black. Abbreviations: ET, endocrine therapy; LCL, lower confidence limit; OR, odds ratio; pCR, pathological complete response; T-DM1, ado-trastuzumab emtansine; UCL, upper confidence limit.

## Data Availability

The datasets analyzed for this manuscript are available from the WSG by reasonable request (www.wsg-online.com).

## Supplementary Materials

The following are available online at https://www.mdpi.com/article/10.3390/cancers13194884/s1, Figure S1: PD-L1 expression on IC and changes from baseline to Cycle 2, Figure S2: Association between *PIK3CA* mutation status and PAM50 subclass, Figure S3: Association between *PIK3CA* mutation status and CD8 changes over time, Figure S4: A, ADAPT Umbrella and B, ADAPT HER2-positive/HR-positive trial designs (reproduced from Hofmann et al. 2013 [open access under CC license]).
