# Supplementary material for: Immune Markers and Tumor-Related Processes Predict Neoadjuvant Therapy Response in the WSG-ADAPT HER2-Positive/Hormone Receptor-Positive Trial in Early Breast Cancer
Source: Cancers (Basel). 2021 Sep 29;13(19):4884. doi: 10.3390/cancers13194884 (PMC8508505; doi:10.3390/cancers13194884)
Supplement: Supplementary file 1 [file cancers-13-04884-s001.zip › cancers-1266160-supplementary.pdf]

# Supplementary Materials: Immune Markers and Tumor-Related Processes Predict Neoadjuvant Therapy Response in the WSG-ADAPT HER2-Positive/Hormone Receptor-Positive Trial in Early Breast Cancer

**Nadia Harbeck, Raquel von Schumann, Ronald Ernest Kates, Michael Braun, Sherko Kuemmel, Claudia Schumacher, Jochem Potenberg, Wolfram Malter, Doris Augustin, Bahriye Aktas, Helmut Forstbauer, Joke Tio, Eva-Maria Grischke, Claudia Biehl, Cornelia Liedtke, Sanne Lysbet De Haas, Regula Deurloo, Rachel Wuerstlein, Hans Heinrich Kreipe and Oleg Gluz**

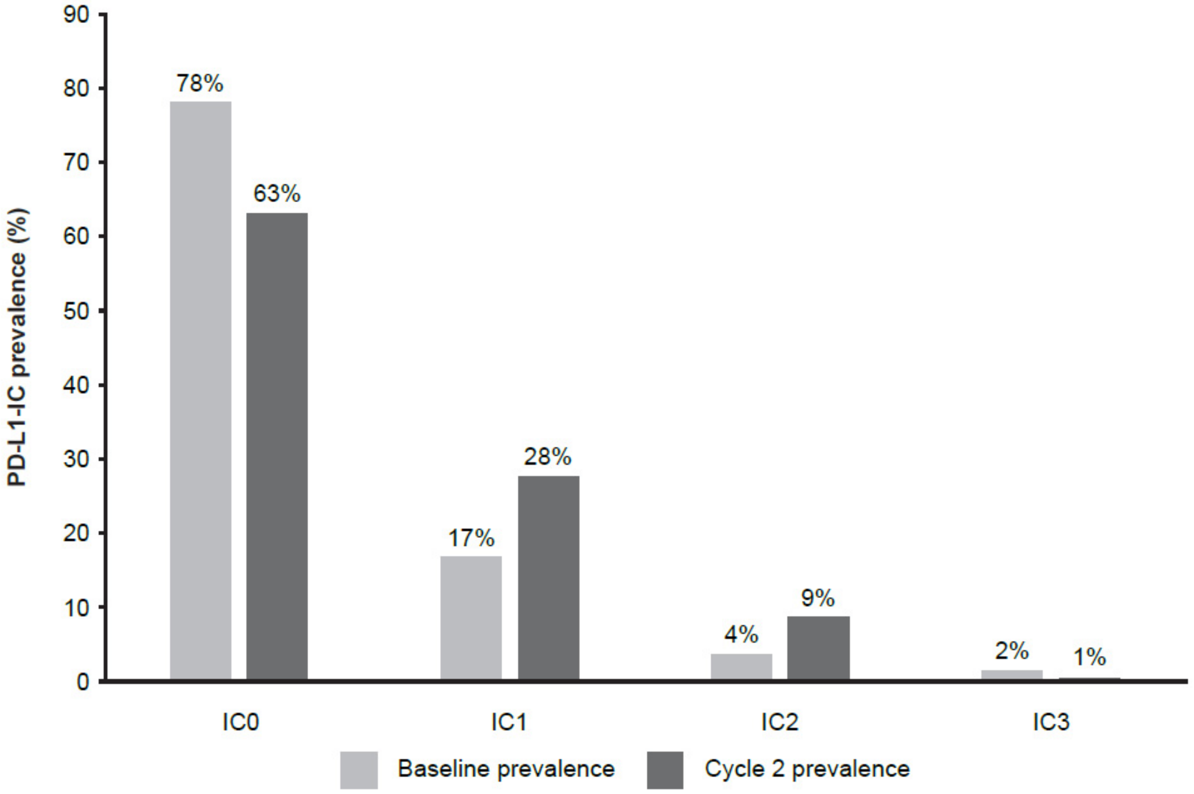


**Figure S1.** PD-L1 expression on IC and changes from baseline to Cycle 2. IC0, IHC staining on <1% of tumor area; IC1, ≥1% and <5%; IC2, ≥5% and <10%; IC3, ≥10%. IC, immune cells; IHC, immunohistochemistry; PD-L1, programmed death-ligand 1.

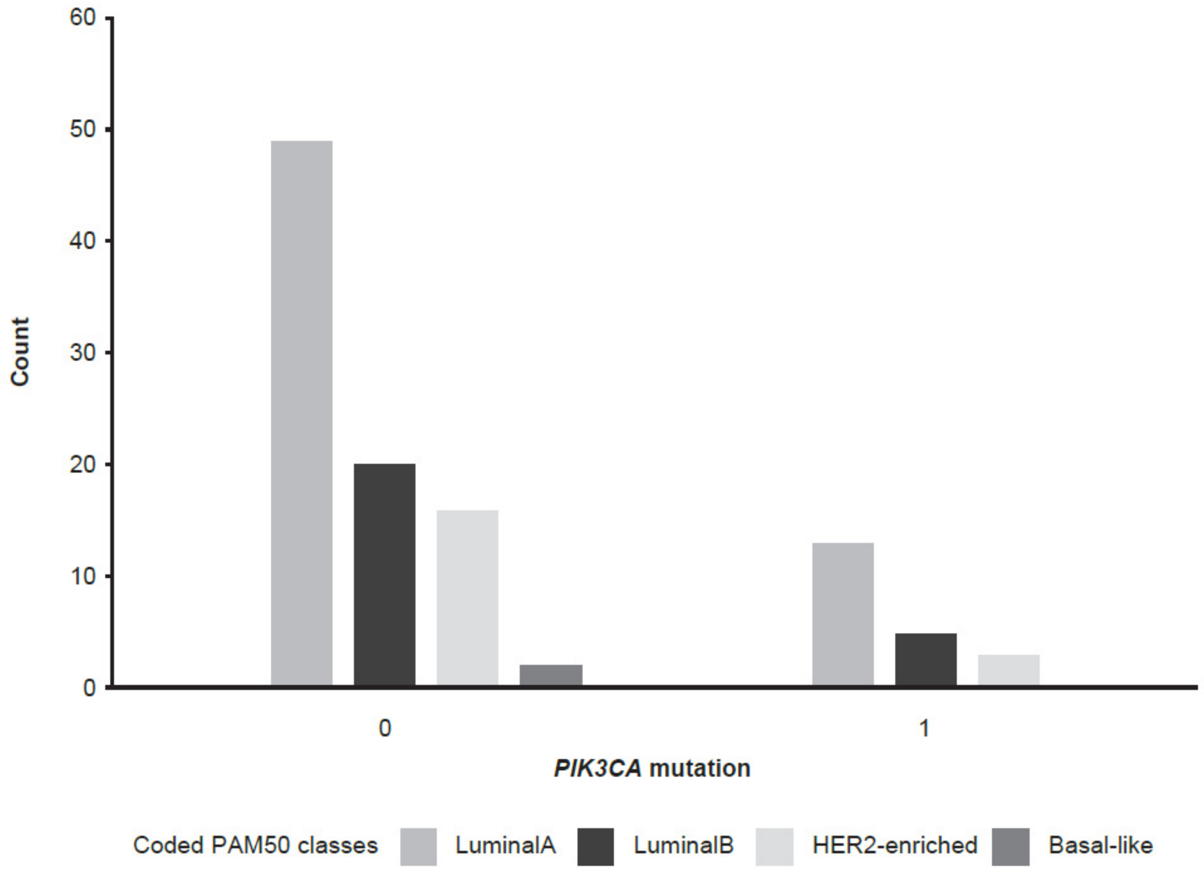


**Figure S2.** Association between *PIK3CA* mutation status and PAM50 subclass.

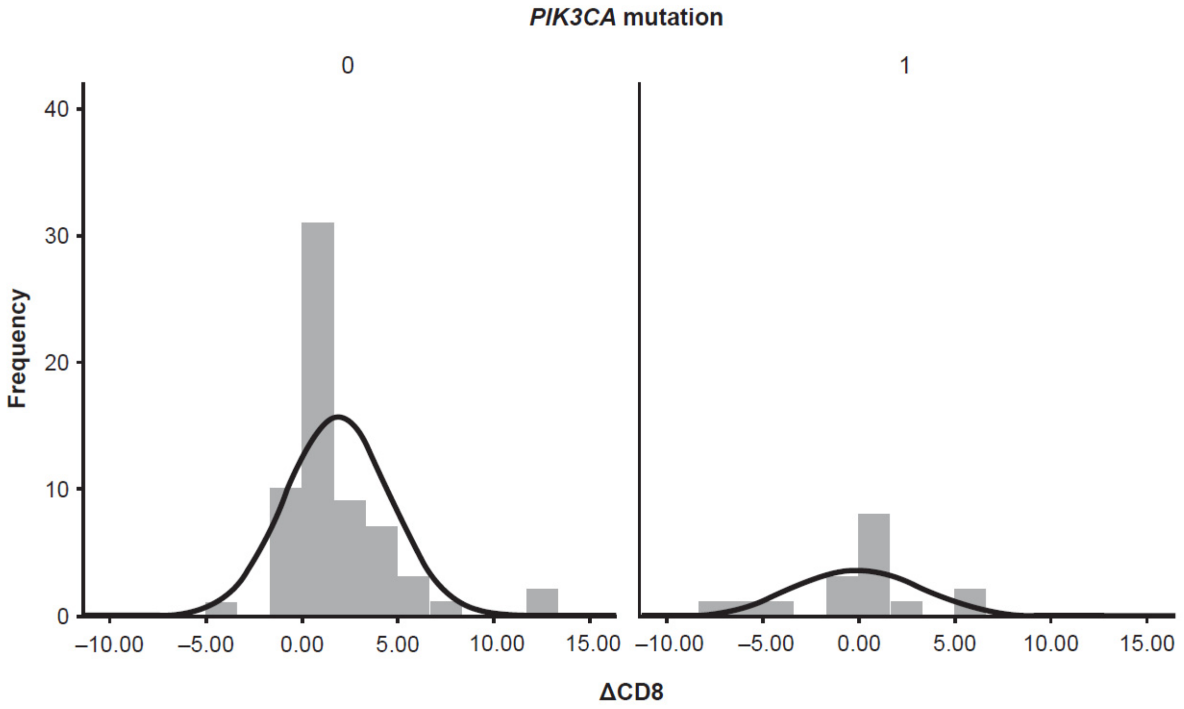


**Figure S3.** Association between *PIK3CA* mutation status and CD8 changes over time.

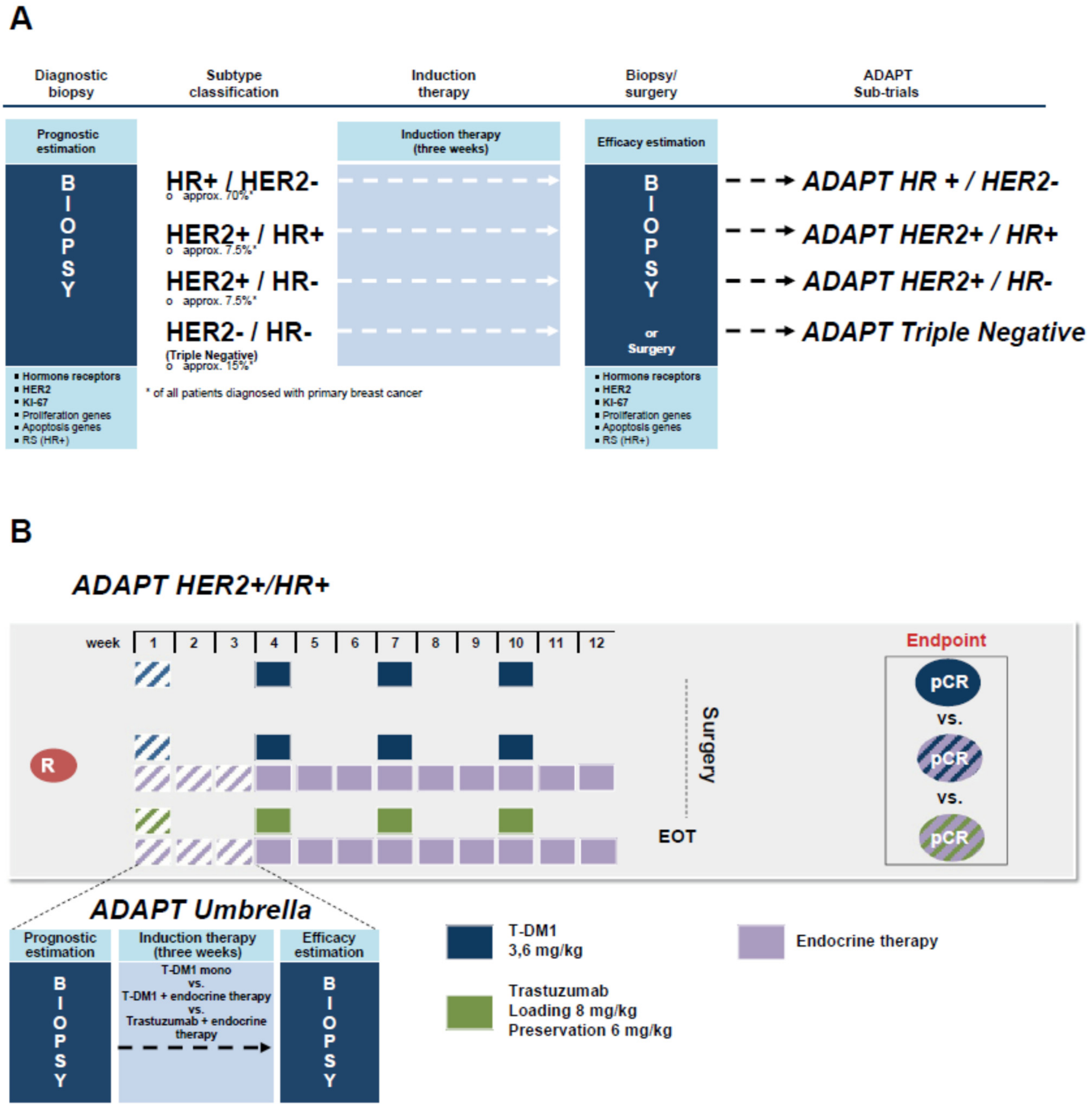


**Figure S4.** (**A**) ADAPT Umbrella and (**B**) ADAPT HER2-positive/HR-positive trial designs (reproduced from Hofmann et al. 2013 [open access under CC license]) [48]. RS, recurrence score.
